# A Two-Way Proposal for the Determination of Bioequivalence for Narrow Therapeutic Index Drugs in the European Union

**DOI:** 10.3390/pharmaceutics16050598

**Published:** 2024-04-28

**Authors:** Paulo Paixao, Alfredo Garcia Arieta, Nuno Silva, Zvonimir Petric, Milton Bonelli, José Augusto Guimarães Morais, Kevin Blake, Luís Filipe Gouveia

**Affiliations:** 1 Research Institute for Medicines (iMed.ULisboa), Faculty of Pharmacy, University of Lisbon, 1649-004 Lisboa, Portugal; 2 Methodology Working Party of the EMA Committee for Medicinal Products for Human Use, 1083 HS Amsterdam, The Netherlands; 3 The Spanish Agency of Medicines and Medical Devices, 28022 Madrid, Spain; 4 European Medicines Agency, 1083 HS Amsterdam, The Netherlands

**Keywords:** narrow therapeutic index drugs, bioequivalence, medicines regulation, generic medicinal products

## Abstract

In the European Union, bioequivalence (BE) for narrow therapeutic index (NTI) drugs is currently demonstrated when the 90% confidence interval for the ratio of the population geometric means of the test and reference products for AUC, and in some cases for Cmax, falls within the acceptance range of 90.00% to 111.11%. However, meeting this requirement results in an increased difficulty of demonstrating BE and a need for clinical trials with larger subject sample sizes, especially for medium-to-high variability drugs. To address this challenge, a scaled average BE based on the reference product within-subject variability for narrowing the acceptance range of NTI drugs was recently proposed. However, this approach showed increased type I error (T1E), especially close to the cut-off point between the unscaled and scaled portions of the method. Based on simulations, this limitation can be overcome by predefining the protocol the path to be followed: either the fixed 90.00–111.11% acceptance range approach or the previously proposed scaled average BE approach with a slight adjustment of the one-sided significance level α to 0.042 for a 2 × 3 × 3 partial replicate design and without a lower cut-off point. This results in a mixed approach allowing to reduce the sample size whilst not inflating the T1E.

## 1. Introduction

Bioequivalence (BE) has long been considered a fundamental way to conclude comparable in vivo performance in terms of safety and efficacy between two orally administered drug products containing the same active moiety. BE is determined by assessing sufficient similarity in the rate and extent of absorption observed when comparing plasma concentration–time curves after administering different drug products to healthy subjects [1]. This assessment of BE is usually conducted by means of the average BE (ABE) approach, which consists of calculation of the 90% confidence interval (CI) for the population geometric mean ratio (GMR) of the test-to-reference product for the pharmacokinetic parameters being assessed. These parameters are normally the area under the plasma drug concentration–time curve (AUC), reflecting the extent of exposure, and the highest (or peak) plasma concentration (Cmax), reflecting the absorption rate or peak exposure [2]. BE is confirmed if the 90% CI for the GMR falls within the acceptance limits, typically considering that a 20% difference should not result in clinically relevant differences [3].

For some drugs, however, a slight change in the administered dose could lead to significant therapeutic shortcomings or adverse drug reactions [4]. For these narrow therapeutic index (NTI) drugs, a more conservative difference of up to 10% is usually considered to be clinically acceptable. Due to this, the European Medicines Agency (EMA) currently requires the BE of these NTI drugs to be decided based on a tighter acceptance range of 90.00–111.11% [5]. This narrower limit results in an increased difficulty to demonstrate BE and requires a larger sample size to show BE, making these types of drugs less attractive for the generic industry and potentially resulting in fewer treatments available for the patients.

Recently, an alternative, less arbitrary approach has been proposed for assessing the BE of NTI drugs by the current co-authors which would allow for a lower number of subjects required to show BE without an expected increase in clinical risk [6]. This consists of an ABE with Narrowed Limits based on the Intra-subject Variability of the Reference product (NLIVR), following a similar approach to widening the acceptance range of Cmax for highly variable drug products (HVDPs) [5]. This NLIVR criterion was initially proposed for within (intra)-subject coefficient of variation (WSCV) values ranging from approximately 14% to 30%. For WSCV values lower than 14%, the tighter acceptance range of 90.00–111.11% was to be used. For WSCV values higher than 30%, the normal acceptance range of 80.00–125.00% was to be used. However, similar to the HVDP criterion, this approach showed a slight increase in type I error (T1E), particularly at the lower cut-off value of the narrowed limits [7], where a T1E increase of up to 7% was observed. This is due to the fact that since the BE limits themselves become random variables, there is a risk of misclassification due to uncertainty in the reference product variability determination. This is particularly evident in the range of WSCV values where the scaled method starts to be applicable [8]. T1E is defined as the probability of erroneously claiming equivalence between test and reference products, and since this is generally assumed to be only 5%, a BE criterion that results in an increase in this T1E is generally seen as a matter of concern [9].

**Figure 1:**
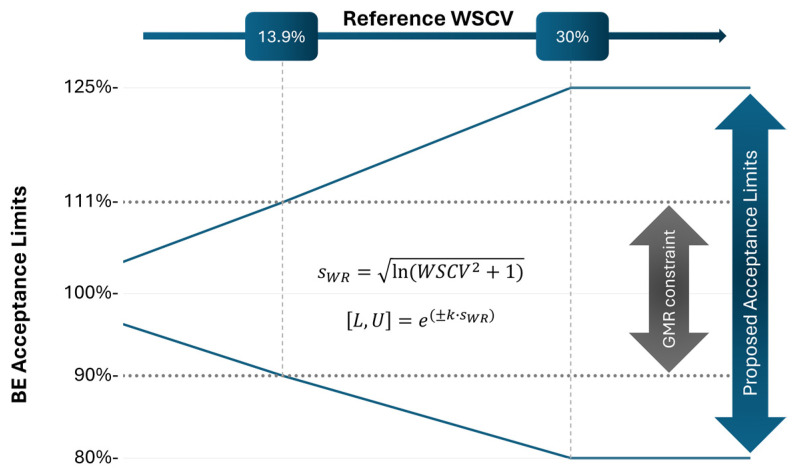
Proposed acceptance limits for the GMR CI of NTI drugs according to the WSCV of the reference product.

In this work, we aimed to address the limitations of our initial NLIVR approach by implementing two key modifications. Firstly, we removed the lower cut-off value which previously defined the use of a fixed 90.00–111.11% acceptance range for low WSCV values and a scaled approach for higher WSCV values. Secondly, we adjusted the significance level to further mitigate the increase in T1E. A graphical representation of the current strategy is shown in Figure 1. Additionally, we evaluated the effect of these changes on the performance for determining BE in products containing NTI drugs using power curves simulated under various assumptions, conditions, and sample size requirements, all in a 2 × 3 × 3 partial replicate design. Based on this, a mixed approach allowing to reduce the sample size whilst not inflating T1E is finally presented. Furthermore, while it is presented here for the purpose of NTI drug BE assessment, it may be noted that this approach can also be considered for HVDPs, with a similar performance expected.

## 2. Materials and Methods

R version 3.6.2 (Platform x86_64_264-mingw32/x64, 2019, The R Foundation for Statistical Computing) [10] was used for simulations on a PC with an Intel Core i7-1165G7 processor and 16 Gb RAM. The package PowerTOST [11] for the statistical language R was used for all calculations.

### 2.1. Type I Error

For the estimation of the T1E (also sometimes referred to as consumer’s risk in clinical trials) for studies with a two-treatment, three-sequence (TRR-RTR-RRT), three-period (2 × 3 × 3) partial replicate design, the number of subjects in the simulations ranged from 9 to 114 (in increments of 3 subjects), and the WSCV value of the reference product varied from 5% to 40% (in increments of 0.125%). The function power.scABEL, incorporating a user-defined reg_const additional function to characterize the proposed narrow therapeutic index (NTI) criterion, was utilized in the PowerTOST package. In this case, the criterion considers that the acceptance range for WSCV values between 0% and 30% is defined by [U, L] = exp [±k·s_WR_], where k is a regulatory constant with a value of 0.76 and s_WR_ is the standard deviation that corresponds to the within-subject variation of the reference product [6]. For WSCV values above 30% (s_WR_ = 0.29356), the 80.00–125.00% acceptance range was applied. One million BE studies were simulated in each of these individual scenarios of number of subjects/WSCV both with and without the additional constraint of the GMR inside the 90.00–111.11% range. Given a GMR just above the defined acceptance limit, and with the corresponding confidence interval (CI) always positioned outside the acceptance range by definition, it would inevitably lead to a non-BE conclusion. Therefore, the probability of concluding bioequivalence should consistently remain below 5%. When that is not the case, and a higher probability is observed, T1E inflation is observed. Thus, for the current approach, as shown in Figure 1, the GMRs were changed depending on the WSCV of the reference product according to the following:

GMR = *e*^−0.76s_WR_^ if WSCV_reference_ < 0.30, (1)

GMR = 0.80 if WSCV_reference_ ≥ 0.30, (2)

i.e., at the lower limit of the acceptance range and under the assumption of homoscedasticity (WSCV of test = WSCV of reference). Based on the binomial test with this number of simulations, an empirical T1E rate above 0.05036 was considered statistically significantly inflated [12]. Initially, the one-sided significance level α for calculating the CIs was set to a value of 0.05, as conventionally required by regulatory agencies. Subsequently, this value was empirically reduced to 0.042 in order to limit the final T1E to be less than 0.05.

### 2.2. Power Analysis

To construct the overall power curves, a similar protocol to the T1E analysis considering the additional constraint of a GMR inside the 90.00–111.11% range was performed. However, fixed geometric mean ratios of 0.95, 0.925, 0.90, and 0.85 (representing real differences of 5%, 7.5%, 10%, and 15%) were assumed. The power results represent the percentage of studies showing BE in each scenario. The one-sided significance level α for calculating the CIs was also set to a value of 0.042.

### 2.3. Sample Size

The sample size is determined through iterative assessment of the power of the TOST procedure, gauged by the success rate of demonstrating bioequivalence (BE) as the sample size increases. To calculate the sample size for a BE trial, several factors need to be defined: (a) the one-sided significance level α, set at 0.05 or 0.042; (b) the type II error, β, which determines the trial’s power (1-β); (c) the BE acceptance range; (d) the expected GMR of the BE metrics; and (e) the WSCV, associated with the variability within individuals for the reference product. For the current EMA criterion, the acceptance range is the present regulatory tighter limits, defined as 90.00 to 111.11%. For the proposed approach, the acceptance range for WSCV values between 0% and 30% is defined by [U, L] = exp [±k·s_WR_], and for WSCV values above 30%, the fixed acceptance range of 80.00–125.00% is applied. Again, an additional constraint of the GMR inside the 90.00–111.11% range is also considered. In this study, the GMRs were changed across scenarios ranging from no difference between both products, with the anticipated GMR of the bioequivalence (BE) metrics set at 1, to escalating real differences of 2.5%, 5%, and 7.5% (corresponding to GMRs of 0.975, 0.95, and 0.925, respectively), with a power level of 80% or 90%. The WSCV was varied incrementally from 5% to 40%, adjusting in steps of 0.125%. The same within-subject variability was assumed for the test and reference products; however, when needed, s_WR_ was estimated only from the data of the reference product. The study design in all scenarios was a two-treatment, three-sequence (TRR-RTR-RRT), three-period (2 × 3 × 3) partial replicate design. The sample sizes for the current EMA NTI drug BE criterion were computed using the ‘Exact’ method within the sampleN.TOST function of the PowerTOST package. These calculations utilize formulas employing Owen’s Q method [13]. The sample sizes for the proposed criterion were estimated through simulations utilizing the sampleN.scABEL function within the PowerTOST package as previously described, incorporating the user-defined reg_const function [11]. Each computed sample size represents the minimum number of subjects required to achieve the target power, assuming an equal distribution of subjects across sequences. In essence, the power results were rounded upwards, ensuring that at the specified sample size, the achieved power was at least as high as the stated level.

## 3. Results

### 3.1. Type I Error

**Figure 2:**
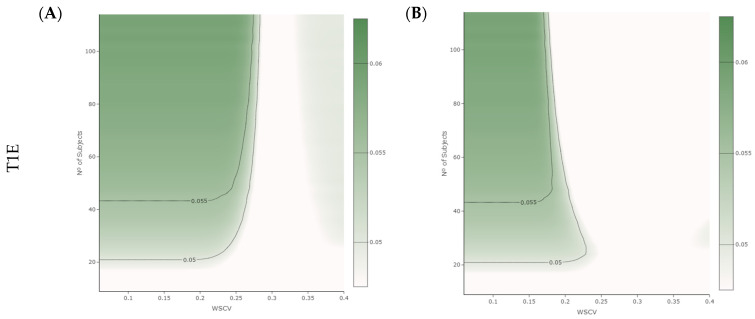
T1E for the proposed NLIVR conditions (without the lower cut-off point between the unscaled and scaled portions) without (**A**) and with (**B**) a GMR constraint for different WSCV values and numbers of subjects.

The results from the T1E analysis using a one-sided significance level α of 0.05 for calculating the CI are presented in Figure 2. Figure 2A shows the ability to conclude BE at the lower limit of the acceptance range for specific WSCV values of the reference product and sample size considering the proposed NLIVR criterion without the lower cut-off value of the narrowed limits. Figure 2B shows the same relationship for the NLIVR criterion but with the additional constraint of the GMR falling within the 90.00–111.11% range, as proposed previously [7]. In both situations, there is a slight inflation in T1E in the region where the BE limits for scaled average BE are narrowed proportionally to the variability. The T1E value is always below 6%, and it is not observed for variation values close to and higher than 30%, where the maximum acceptance range is fixed at 80.00–125.00%. Incorporating the GMR constraint did not change the highest inflation of the T1E, but it clearly reduced the overall area of T1E inflation (Figure 2B). Most significantly, by empirically changing the one-sided significance level α of the CIs to 0.042, the T1E became lower than 5% in all the studied conditions.

### 3.2. Power Analysis

**Figure 3:**
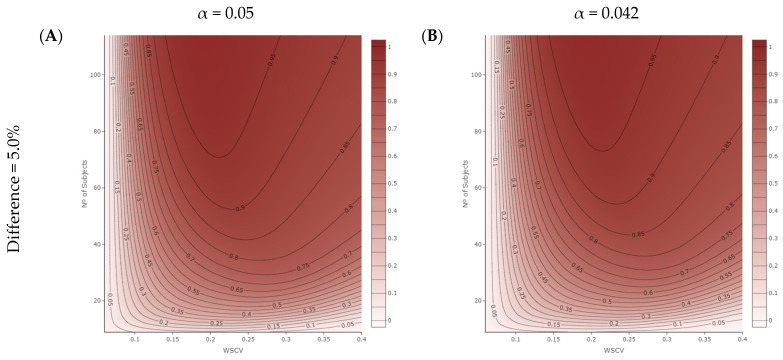

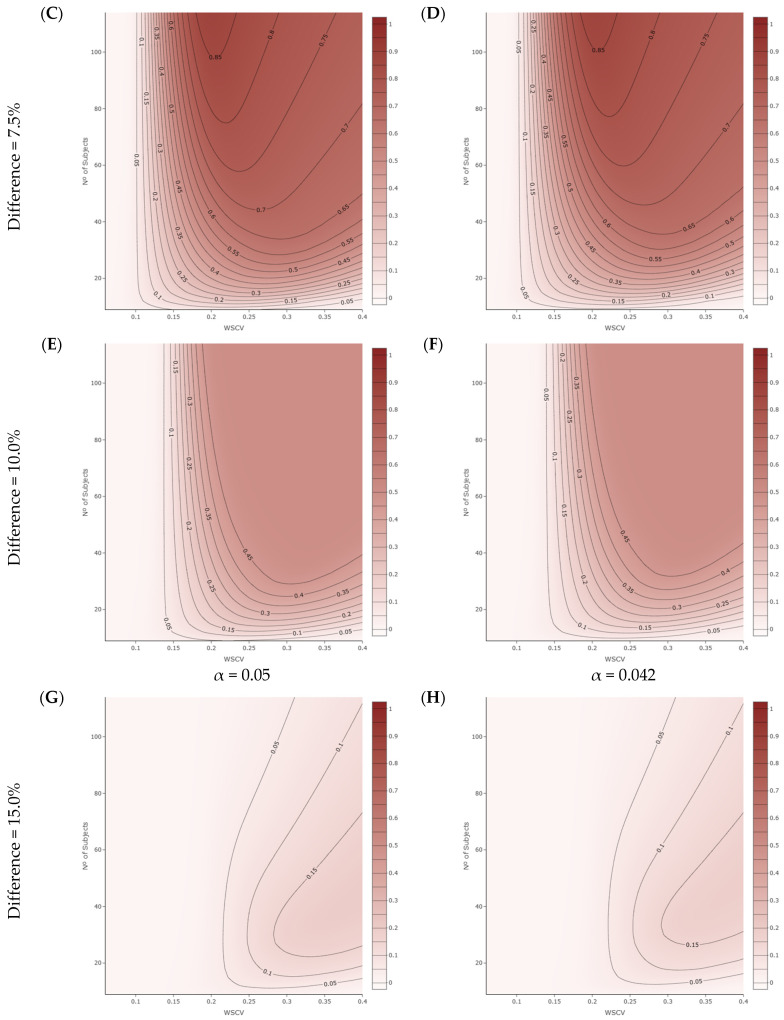
Power analysis for the proposed NLIVR conditions with a GMR constraint, assuming increasing nominal true differences between the test and reference products, for different WSCV values and numbers of subjects and for α = 0.05 (Figures (**A**,**C**,**E**,**G**)) or α = 0.042 (Figures (**B**,**D**,**F**,**H**)). The legend represents the probability of concluding BE.

The results of the power analysis are displayed in Figure 3. The left plots (Figure 3A,C,E,G) illustrate the power to conclude BE for specific products with real differences of 5%, 7.5%, 10%, and 15% as a function of the WSCV of the reference product and the number of subjects included in the simulated BE trial. These plots represent the proposed NLIVR criterion without a lower cut-off value between the unscaled and scaled portions of the narrowed limits and with the constraint of the GMR falling within the 90.00–111.11% range. The right plots (Figure 3B,D,F,H) illustrate the same relationship for the same NLIVR criterion but with the one-sided significance level α set to 0.042 for calculating the CI. It can be seen that since no lower cut-off value was imposed and, contrary to our previous NLIVR proposal [6,7], no fixed 90.00–111.11% limits were considered, the probability of concluding BE in the product performance differing by less than 10% was very limited (less than 5%) for the lower WSCV values (Figure 3A,C). A product differing by 7.5% from the reference would not be considered to have BE if the WSCV of the reference product was lower than approximately 10% thanks to the use of a conservative regulatory “proportionality” constant k, which was set to 0.760. However, consistent with the findings from the previous NLIVR proposal, the probability of concluding BE greatly increased with an increase in the WSCV and the number of subjects, as also expected based on the direct relationship between the acceptance range and the WSCV. The likelihood of concluding BE could be notably high, reaching up to 95% for products with only a 5% difference (Figure 3A). However, this probability diminished considerably as the theoretical difference between products neared the 20% threshold, consistently falling below 5% (plot not displayed). Indeed, even with a true difference of 10% between the two products (Figure 3E), the highest probability of concluding BE was only around 50%, primarily due to the application of the GMR constraint. If the difference between products increased, as seen in Figure 3G, this added to the difficulty in concluding BE, as the increase in the sample size could indeed result in a loss of power to conclude BE. The change in the one-sided significance level to a value of 0.042 did not significantly alter the power curves of the method, resulting in only a very slight overall shift to the right (Figure 3B,D,F,H).

### 3.3. Sample Size

**Figure 4:**
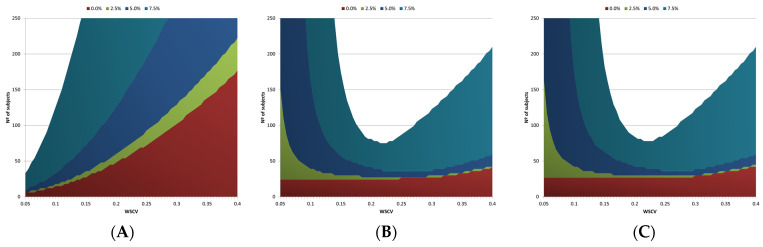
Sample sizes for the EMA’s current NTI drug criteria (**A**) and the proposed NLIVR conditions with the GMR constraint and α = 0.05 (**B**) or α = 0.042 (**C**) for 80% power and assuming a true difference ranging from 0% to 7.5%.

The subject sample size requirements for achieving 80% power to conclude bioequivalence (BE) are outlined. These requirements were compared across different criteria: the current EMA NTID fixed acceptance range of 90.00% to 111.11% (Figure 4A and Table 1), the proposed NLIVR criterion without the lower cut-off value but with the GMR maintained within the 90.00% to 111.11% constraint (Figure 4B and Table 1), and the same NLIVR criterion with the one-sided significance level α set to 0.042 (Figure 4C and Table 1). The sample size requirements to conclude BE with 90% power under the same conditions can also be seen in Table 1. Comparing the current NTI drug criterion from the EMA, which has a fixed acceptance interval, with the NLIVR criterion in all studied situations revealed that the EMA criterion demands fewer subjects when the WSCV values are lower than approximately 14%. However, as the WSCV values increase, the NLIVR approach consistently requires a smaller sample size. If, by the current NTID criterion, concluding BE between two equal products, with a GMR = 1, would require a significant number of subjects for a higher WSCV value (Figure 4A), the NLIVR criterion would make the approval of two products with a real difference of up to 7.5% (still considered similar under the currently acceptable 10% difference) much more demanding at WSCV values lower than 14% (Figure 4B). Again, changing the one-sided significance level of the CIs from α = 0.05 to α = 0.042 had no major consequences, resulting in only a slight increase in the sample size requirements (Figure 4C).

## 4. Discussion

The proposed scaled ABE approach for NTI drugs with an acceptance range proportional to the WSCV of the reference product and a slight adjustment of the one-sided significance level α to 0.042, resulting in the calculation of the 91.6% CI, can reduce the sample size required to show BE whilst controlling T1E. This contrasts with the previously proposed criterion [6] with an additional constraint of the GMR contained within a 90.00–111.11% acceptance range [7] where the sample size of the BE studies was reduced but an undesirable T1E increase was observed, particularly closer to the lower cut-off point of the BE acceptance limits that were scaled in relation to the WSCV.

This inflation in T1E due to either underestimation or overestimation of the WSCV was anticipated as it is known, for similar methods, to be influenced by many factors such as the regulatory constant value, the cut-off point between the unscaled and scaled portions, and the existence of continuity at the cut-off point [14]. Therefore, in the current simulations, we conducted tests involving removal of the lower cut-off point in a two-treatment, three-sequence (TRR-RTR-RRT), three-period (2 × 3 × 3) partial replicate design. As shown in Figure 2A, T1E inflation still occurred in the majority of the WSCV scenarios where scaling was accepted, in line with the fact that the used WSCV is only an estimation of the true WSCV of the reference product [12,15,16]. However, in contrast to the original proposal with the lower cut-off [7], the T1E inflation was basically homogenous, with a slight increase noted with larger sample sizes. This inflation consistently remained below 6% across all the study conditions. Furthermore, the incorporation of the GMR constraint did not reduce the maximum T1E inflation (Figure 2B), but it notably reduced the area of T1E inflation to be contained within WSCV values lower than 18%, especially for larger sample sizes. This occurred since, at higher variabilities, the GMR constraint becomes the primary determinant of BE conclusion [8]. The inflation in T1E represents an undesirable outcome, indicating deficiencies in the employed statistical methods. Consequently, several approaches have been proposed in order to address this issue [12,17,18,19]. One potential solution involves adjusting the significance level of the statistical test using an adjusted coverage probability for the CI calculation, which will be higher than 90%. In the present context, given the homogenous increase in the T1E values, the use of the one-sided significance level α = 0.042 for calculating the corresponding 91.6% CIs consistently resulted in T1E values below the acceptable 5% threshold in all the studied scenarios. This simple adjustment obviates, for example, the need for an iterative process of optimization for an α penalty [12].

The overall performance of the proposed regulatory criterion without the lower cut-off value is illustrated in Figure 3. Since the narrowing of the acceptance interval was continuous for WSCV values ranging between 0% to 30%, a drug product differing by 5% from the reference was considered as not bioequivalent for WSCV values lower than around 6.5%, regardless of the number of subjects included in the clinical trial (Figure 3A). When compared to the EMA’s current NTI drug criteria, this approach resulted in a larger sample size, as the fixed acceptance limits of 90.00–111.11% allow for BE conclusion for a product differing by up to 10%, but this is in line with the expected performance of the current regulatory criteria of the FDA for NTI drugs [20]. However, as the WSCV values increase, the power to conclude BE also increases, as previously demonstrated [7,21]. Similar trends were observed in the remaining scenarios, albeit with a reduced overall probability of concluding BE. In fact, the probability of accepting BE between products differing by 10% peaked at a maximum of around 50% (Figure 3E), primarily due to the requirement that the GMR should fall within the 90.00–111.11% acceptance range [7,22]. Additionally, it is noteworthy that adjusting the one-sided significance level to α = 0.042 (Figure 3B,D,F,H) did not significantly alter the probability curves for accepting BE between products. As a result, the overall power remains quite similar.

**Figure 5:**
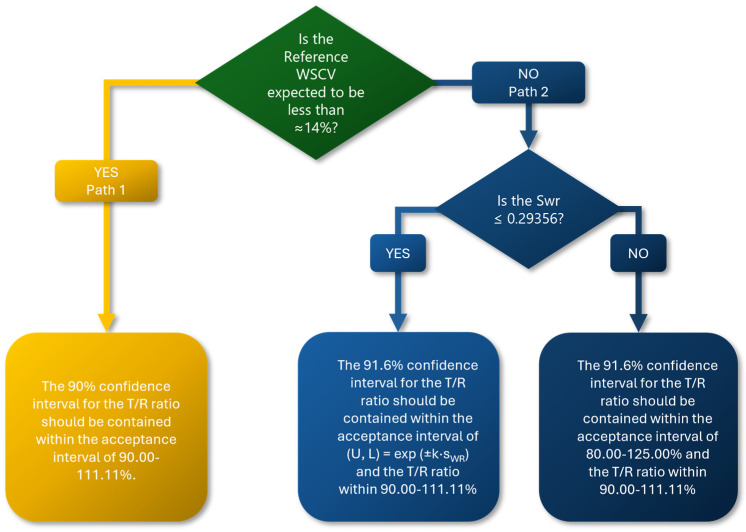
Decision tree for the proposed assessment of the BE for NTI drugs.

The current EMA NTI drug criteria, which employ a fixed 90.00–111.11% acceptance range, impose an acceptable mean difference of less than 10% between two products. This value aligns with the acceptable maximum 10% difference of the assay between batches in the routine batch release (±5%) of the final (reference) product, prompting questions about the necessity for further narrowing the acceptance criteria below these values. However, the advantages of narrowing (from the normal 80.00–120.00% acceptance criteria) the acceptance range based on the reference WSCV values become evident as the variability increases. Therefore, as previously proposed [6,7], we suggest that both approaches are valid and complementary to one another. In this case, and in order to remove the observed extreme T1E inflation observed around the cut-off value of WSCV = 13.93%, applicants must choose their path prior to starting the clinical trial: either the current EMA NTI drug criteria (i.e., Path 1), if anticipating low WSCV variability, or the newly proposed NLIVR approach without the lower cut-off value (i.e., Path 2), if anticipating medium-to-high WSCV variability, following the proposal outlined in Figure 5. However, it could be criticized that this strategy would result in the approval of different generics based on different standards, leading to different levels of assurance on the clinical equivalence for the patients. In this line of reasoning, another possible alternative could be that the regulatory agencies predefine in product-specific BE guidelines the approach to be followed for the generics of each reference product according to the expected WSCV.

It should again be highlighted that in order to have a BE statistical analysis under the presently proposed criteria and without T1E inflation, the sample size optimization in these BE studies would have mixed behavior, as can be seen in the plots from Figure 4. If the applicant or the regulatory authority chooses the classical path of the fixed tight acceptance range of 90.00–111.11%, the sample size required for demonstrating BE will be less than 27 subjects (in a 2 × 3 × 3 partial replicate design with two identical products) for WSCV values below, e.g., 14%. However, if the expected WSCV is higher, choosing the alternative NLIVR approach would result in increased statistical power to demonstrate BE and a corresponding significant reduction in the required number of subjects. However, as the choice of path must be made prior to the clinical trial, if Path 2 is chosen and the observed WSCV is low, then it will be more difficult to demonstrate BE as the acceptance range may be narrower than the 90.00–111.11% range of Path 1, and vice versa. Therefore, to ensure applicability, even though different paths can be chosen for different parameters (e.g., AUC and Cmax) or for different analytes (e.g., in fixed-dose combinations), the decision should be predefined in the protocol or in the product-specific BE guideline. In this regard, the data from Table 1 can be of added value for applicants and regulators in deciding the path to be followed for an NTI drug product.

There is significant interest in harmonizing the standards and criteria for assessing BE worldwide. This would simplify the development and approval of drugs in different regulatory areas, with evident economical and drug accessibility benefits for both patients and the pharmaceutical industry. In this regard, a guidance document from the International Council for Harmonisation of Technical Requirements for Registration of Pharmaceuticals for Human Use (ICH) is currently in development, the M13 guideline on BE for Immediate-Release Solid Oral Dosage Forms, where the topic of BE assessment for NTI drugs will be addressed in its Tier 3 moment, expected to occur in late 2024 [23]. The presented criteria for the assessment of BE between two NTI drug products in the current study propose a mixed approach that combines the possibility of using either a fixed 90.00–111.11% acceptance range or a scaled one for concluding BE. These two individual approaches are currently in use in different regulatory areas. For example, the EMA uses the fixed acceptance range of 90.00–111.11% [5], while the FDA uses a reference-scaled approach [24]. For direct comparison to the FDA, our suggested proposal is yet to be tested with a full replicate four-period study design. This is due to the fact that the FDA criteria also consider a criterion that compares the variability between the test and reference products, which implies the need for a four-period design. That is indeed an additional requirement which increases the ability to discriminate between two products [25], but the actual relevance of which is still not known (e.g., if products may fail to show equivalence with respect to the WSCV with the present acceptance criterion). Without delving into the debate about the advantages of comparing products’ variability, the presently proposed approach is part of ongoing efforts toward future discussions on the much needed harmonization in this aspect which, due to its many similarities with the current HVDP approaches, may also be easily expanded to that field too.

## 5. Conclusions

The use of scaled average BE based on the reference product variability to narrow the confidence limits for NTI drugs is an alternative concept to the current EMA criteria involving a fixed acceptance range of 90.00–111.11%. It results in a substantial reduction in the required subject sample sizes in clinical trials for medium-to-high variability NTI drugs. However, as with similar methods already in use for HDVP BE assessment, this results in an increase in T1E, especially if a cut-off point between the unscaled and scaled portions of the method is considered. By proposing a prior and independent two-way choice proposal, the applicants can decide to employ either the fixed interval approach or the continuous variability scaled approach with a minor adjustment of the one-sided significance level, leading to the calculation of a 91.6% CI for a 2 × 3 × 3 partial replicate design. This mixed-choice approach offers a versatile solution that reduces the required subject sample sizes when relevant and without any expectable increase in the clinical risk, all without the undesirable increase in T1E.

## Figures and Tables

**Table 1:** Sample sizes for the EMA’s current NTI drug criteria and for the proposed NLIVR conditions with the GMR constraint at α = 0.05 or α = 0.042 in a 2 × 3 × 3 partial replicate study design for a power of 80% and 90%, and assuming a GMR ranging from 1.000 to 0.925.

			EMA NTI				NLIVR a = 0.05				NLIVR a = 0.042			
	CV	GMR	1.000	0.975	0.950	0.925	1.000	0.975	0.950	0.925	1.000	0.975	0.950	0.925
**80% Power**	5%		6	6	9	33	24	153	-	-	27	165	-	-
	10%		15	18	33	126	24	39	162	-	27	42	174	-
	15%		27	36	72	276	24	30	57	174	27	33	63	183
	20%		48	60	126	486	24	27	42	81	27	30	42	84
	25%		72	93	195	750	27	27	36	87	27	30	36	87
	30%		102	129	276	1068	27	30	36	123	30	30	39	123
	35%		138	174	369	1431	33	33	45	162	36	36	48	162
	40%		177	222	474	1836	42	42	60	210	42	45	60	210
**90% Power**	5%		6	6	12	45	30	210	-	-	33	222	-	-
	10%		18	21	45	171	30	54	222	-	33	57	237	-
	15%		36	48	99	384	30	39	81	243	33	42	84	255
	20%		60	81	174	672	30	36	54	129	33	39	60	129
	25%		93	123	270	1041	33	36	54	189	33	36	54	189
	30%		129	177	381	1476	36	39	69	267	36	39	69	267
	35%		174	234	510	1980	45	51	93	357	45	51	93	357
	40%		222	300	654	2541	57	63	120	459	57	63	120	459

## Data Availability

The data presented in this study are available on request from the corresponding author.
